# Proactive and creative personality as predictors of student engagement and innovative behavior among Chinese university students

**DOI:** 10.3389/fpsyg.2026.1752174

**Published:** 2026-03-02

**Authors:** Wen Yu Meng, Kab Won Kang, Chun Lian Quan

**Affiliations:** 1 School of Marxism, Jiangsu Vocational College of Finance and Economics, Huai'an, China; 2 Graduate School of Education, Daejin University, Pocheon, Republic of Korea; 3 Department of Education, Graduate School, Jungwon University, Goesan, Republic of Korea

**Keywords:** Chinese student, creative personality, innovative behavior, proactive personality, student engagement

## Abstract

This study examined the structural relationships among proactive personality, creative personality, student engagement, and innovative behavior among Chinese university students. Specifically, it investigated how proactive and creative personality traits independently and jointly influence student engagement and innovative behavior, while controlling for their mutual effects. Data were collected from multiple universities in China and analyzed using structural equation modeling with maximum likelihood estimation. Results revealed that proactive personality had a significant positive effect on student engagement, whereas creative personality did not. Both proactive and creative personalities significantly influenced innovative behavior, with creative personality showing a stronger effect. Together, proactive and creative personality traits explained the majority of the variability in student engagement and innovative behavior. These findings suggest that proactive personality serves as a strong antecedent of student engagement, while both proactive and creative personalities are key drivers of innovative behavior. Theoretically, the findings reinforce that creativity may not directly enhance engagement but plays a crucial role in fostering innovation. Practically, strengthening proactive personality may enhance students’ academic engagement, whereas developing both proactive and creative personalities may concurrently promote creativity and innovation. In light of the Fourth Industrial Revolution, universities should implement strategies that nurture proactive and creativity-oriented traits among students.

## 1 Introduction

In recent years, the employment rate of Chinese university graduates has become a major concern. According to Zhaopin Limited (2025), the employment rate of college graduates in 2024 was only 55.5%, highlighting a serious employment challenge. Under such circumstances, it is increasingly important for Chinese college students to succeed academically and develop the creative and innovative capabilities demanded by the Fourth Industrial Revolution. In response, the Chinese government has launched various initiatives, such as the College Student Innovation and Entrepreneurship Programs.^1^ Within this context, examining student engagement (SE), innovative behavior (IB), proactive personality (PP), and creative personality (CP), both of which may influence SE and IB among Chinese university students, has become particularly meaningful and timely.

SE has been defined in multiple ways: as the time and effort students invest in learning activities (Tyler, 1949; Pace, 1984; Astin, 1984); as the intensity and quality of participation in learning (Fredricks et al., 2004); and as a state of active, sustained involvement in academic tasks (Schaufeli et al., 2002). SE is widely recognized as a strong predictor of academic performance and school satisfaction (Zhang, 2019).

The concept of IB originates from the concept of “innovation” proposed by the German economist Schumpeter, defined as the development of new products, processes, markets, or organizational forms (Lazzarotti et al., 2011). From an organizational perspective, Hurt et al. (1977) reconceptualized innovation as the individual’s willingness to introduce change and engage in behaviors that generate and implement new ideas (Gu and Peng, 2010).

In higher education, IB refers to students’ intentions and actions to apply novel ideas, knowledge, or perspectives to academic and real-life contexts (Mei et al., 2015) and to enhance group performance through innovative activities (Xin, 2016). While IB in the business context focuses on employees generating and implementing ideas for organizational development, in universities, it centers on students’ efforts to develop and apply new ideas to solve academic and everyday problems (Zhang, 2019).

IB is valuable not only for academic success but also for developing personal problem-solving skills (Zhang, 2019). Understanding which psychological variables influence SE and IB can inform educational programs and interventions aimed at enhancing these outcomes. Among these psychological factors, PP and CP are particularly meaningful.

PP, first conceptualized by Bateman and Crant (1993), is a stable trait reflecting an individual’s tendency to initiate change, be future-oriented, and act persistently toward desired goals (Bateman and Crant, 1993). It has been described as a disposition to take immediate and sustained action (Seibert et al., 1999) and to overcome obstacles to achieve objectives (Ye, 2007). Individuals with strong proactive tendencies are more likely to succeed in their careers (Seibert et al., 1999; Seibert et al., 2001) and, among students, demonstrate higher levels of innovative behavior, self-efficacy, and academic achievement (Kim et al., 2009). Because proactive individuals are self-directed and goal-oriented, PP has been identified as a positive predictor of SE (Wang F., 2013; Wang X. D., 2013; Gao et al., 2015; Li, 2016; Li, 2018).

CP, by contrast, reflects a dispositional tendency toward creative thought and behavior (Guilford, 1950; Wei, 2002; Lin, 2014; Jia et al., 2016). Individuals with a high level of CP are open to novelty, enjoy challenges, and willingly devote time and energy to activities that are intrinsically motivating (Chen and Xiao, 2011; Wang and Wang, 2014). CP facilitates creative thinking and behavior (Wei, 2003) and has been shown to correlate positively with SE (Schaufeli et al., 2002; Zhang et al., 2008). However, such engagement may depend on the learning context: students with high CP are likely to be highly engaged in creative, problem-based, or discovery-oriented learning, but less so in routine or mandatory tasks. This pattern mirrors intelligence research, which exhibits that while intelligence strongly correlates with academic achievement, it is only weakly associated with creativity (Getzels and Jackson, 1962; Benedek et al., 2014). Beyond a certain threshold (e.g., IQ 120), intelligence shows no further association with creativity (Jauk et al., 2013), and the correlation between IB and academic achievement tends to be low (Benedek et al., 2014). These findings suggest that the relationship between PP and SE is likely stronger than that between CP and SE.

Individuals with a PP tend to generate and implement new ideas that drive change (Gu and Peng, 2010) and exhibit a voluntary inclination to adopt innovative behaviors (Scott and Bruce, 1994; Janssen, 2000). Empirical studies consistently show a positive relationship between PP and IB (Crant, 1995; Seibert et al., 2001; Liu, 2013; Cao and Xiang, 2014; Zhang et al., 2016; Pang and Wen, 2017; Mai and Ji, 2019; Zhou et al., 2020).

CP is also closely associated with IB. It reflects an individual’s tendency to engage in creative behavior and influences the production of creative work and outcomes (Feist and Barron, 2003; Wei, 2003; Haller and Courvoisier, 2010). IB, in turn, involves transforming new ideas into practice to promote change (Janssen, 2000; Gu and Peng, 2010) and solving academic or real-life problems through novel approaches (Mei et al., 2015; Xin, 2016; Zhang, 2019). Individuals high in CP often demonstrate pioneering attitudes, resilience, and a willingness to take risks (Schoen et al., 2018). Likewise, those who engage in IB exhibit confidence, responsibility, and persistence despite setbacks (Wang, 2020). Therefore, higher levels of CP are generally associated with stronger IB (Gao and Wang, 2016; Li et al., 2017). Empirical research consistently supports the positive association between CP and IB (Wang F., 2013; Wang X. D., 2013; Li et al., 2014; Song and Gu, 2015; Gao et al., 2016; Jia et al., 2016; Yu and Xiang, 2019).

Based on this literature, this study hypothesizes that PP positively relates to both SE and IB, whereas CP is positively related to IB but may not necessarily influence SE. Even if a positive relationship exists between CP and SE, the relationship between CP and IB is expected to be stronger. Although prior studies have examined these associations individually, few have analyzed them within a single model that considers the mutual or interactive effects of PP and CP. Therefore, this study investigates the structural relationships among PP, CP, SE, and IB, all accounting for the interdependence of PP and CP.

Clarifying the extent to which PP and CP contribute to SE and IB will provide insights into how these traits enhance students’ academic and innovative outcomes. Such understanding may inform educational strategies and program design aimed at promoting SE and IB. Moreover, given China’s collectivist and system-oriented sociocultural environment, examining these psychological variables among Chinese university students carries important academic and practical implications.

## 2 Research method

### 2.1 Participants

The target population comprised undergraduate students enrolled in four-year universities across China. A total of 618 students were recruited from 25 universities located in nine provinces: Chongqing (3 universities), Heilongjiang (2), Henan (3), Anhui (3), Hubei (2), Jiangsu (3), Jiangxi (3), Guizhou (3), and Guangxi (3). Among the participants, 266 were male (43%) and 352 were female (57%). By academic discipline, 316 students (51%) majored in the humanities and social sciences, whereas 302 students (49%) studied science, engineering, or natural sciences. The sample included 120 freshmen (19.4%), 103 sophomores (16.7%), 143 juniors (23.1%), and 252 seniors (40.8%). Data were collected through a mobile-based survey application between June 27 and June 30, 2022.

### 2.2 Research ethics

This study adhered to the Measures for the Ethical Review of Life Science and Medical Research Involving Human Beings,^2^ formulated in accordance with the Biosafety Law of the People’s Republic of China and the Regulations on the Administration of Human Genetic Resources. These measures emphasize protecting human life and health, safeguarding human dignity, and ensuring the lawful rights and interests of research participants.

According to these measures, studies that do not cause direct harm to the human body and do not involve sensitive personal information or commercial interests may qualify for exemption from ethical review. Examples include research using publicly available, anonymized, or lawfully obtained data that comply with relevant laws, regulations, and ethical standards.

As this study employed an anonymous online survey and obtained informed consent from all participants, it met the criteria for exemption from formal ethical review. All procedures complied with applicable ethical principles and regulatory standards (see questionnaire in Appendix A).

### 2.3 Instruments

The PP scale used in this study was adopted from Yi-Feng Chen et al. (2021). The instrument originated from Bateman and Crant (1993) and was refined by Seibert et al. (1999) into a 10-item, single-factor scale. The CP scale was derived from the Chinese version of the Williams Creativity Scale (China Association of Foreign Service Trades, and Beijing Foreign Enterprise Service Group Co., Ltd, 2003). The original 50-item instrument included four subfactors: challenge (12 items), curiosity (14 items), risk-taking (11 items), and imagination (13 items). To reduce participant fatigue and inattentive responses, negatively worded or redundant items were excluded, and representative items from each subfactor were retained. Specifically, two items each were selected from adventurousness (items 24 and 25), curiosity (items 27 and 34), and risk-taking (items 26 and 42), and three from imagination (items 6, 22, and 32), resulting in a total of nine items used for analysis.

The SE scale was adapted from the Utrecht Work Engagement Scale for Students (UWES-S; Schaufeli et al., 2002), which comprises 14 items across three dimensions: vigor (5 items), dedication (5 items), and absorption (4 items).

The IB scale was developed by Wu (2016), who selected seven items from Danis and Dollinger’s (1998) adaptation of Bagozzi and Foxall’s (1995) 32-item instrument derived from Kirton’s (1976) Adaptation–Innovation Inventory.

Table 1 summarizes the item composition, reliability, and convergent validity of each scale. Reliability was assessed using Cronbach’s alpha (*α*), and convergent validity was evaluated through average variance extracted (AVE) and composite reliability (CR). Cronbach’s alpha values ranged from 0.80 to 0.96, AVE values from 0.75 to 0.85, and CR values from 0.88 to 0.95. All AVE values exceeded the 0.50 threshold, and all CR values surpassed 0.70, confirming satisfactory reliability and convergent validity. The AVE and CR indices were calculated by randomly assigning items into subsets (item parceling) and averaging their scores.

**Table 1:** Composition and reliability of measurement instruments.

Scale		Item number	The numbers of items	Cronbach’s *α*	AVE	CR
PP		1–10	10	0.90	0.71	0.88
SE	Vigor	1–5	5	0.94	0.85	0.95
	Dedication	6–10	5	0.90		
	Absorption	11–14	4	0.87		
	Total		14	0.96		
CP	Adventure	1, 2	2	0.88	0.75	0.90
	Curiosity	3, 4	2			
	Imagination	5, 6, 7	3			
	Challenge	8, 9	2			
	Total		9			
IB		1–7	7	0.92	0.81	0.93

PP, proactive personality; CP, creative personality; SE, student engagement; IB, innovative behavior.

### 2.4 Common method bias test

Since all four measurement scales employed a common 5-point Likert format and data were collected simultaneously, the potential for common method bias (CMB) was examined using Harman’s single-factor test. Principal component analysis, constrained to a single factor and performed without rotation, exhibited that the first factor accounted for 37.52% of the total variance. As this value was below the 50% threshold, CMB was determined not to be a significant concern in this study.

### 2.5 Measurement model validation

Data analyses were conducted using IBM SPSS 26 and AMOS 26, with parameter estimation performed through maximum likelihood (ML) methods. Following Anderson and Gerbing’s (1988) two-step approach, the measurement model was first validated before testing the structural model.

Item parceling was conducted using a random number generator following Matsunaga’s (2008) guidelines. The averaged scores of item parcels were used as observed indicators. The parcel for composition was as follows:

- PP: PP1 = items 2, 5, 8, 9; PP2 = items 1, 3, 6; PP3 = items 4, 7, 10;
- CP: CP1 = items 4, 7, 9; CP2 = items 2, 5, 8; CP3 = items 1, 3, 6;
- SE: SE1 = items 5, 7, 8, 9; SE2 = items 1, 2, 3, 4, 14; SE3 = items 6, 10, 11, 12, 13; and
- IB: IB1 = items 1, 5; IB2 = items 2, 6; IB3 = items 3, 4, 7.

All factor loadings were statistically significant (*p* < 0.001): PP (0.82–0.88), CP (0.87–0.88), SE (0.91–0.95), and IB (0.89–0.90).

Model fit was assessed using indices less sensitive to sample size and model complexity—namely, the Tucker–Lewis Index (TLI), Standardized Root Mean Square Residual (SRMR), and Root Mean Square Error of Approximation (RMSEA) (Browne and Cudeck, 1993; Raykov and Marcoulides, 2000). The findings were as follows: TLI = 0.984, SRMR = 0.0179, and RMSEA = 0.053. According to established criteria (Kline, 1998; Hu and Bentler, 1999), model fit is acceptable when TLI ≥ 0.95, SRMR ≤ 0.08, and RMSEA ≤ 0.08. Therefore, the measurement model demonstrated a satisfactory level of fit.

Discriminant validity was assessed using Fornell and Larcker’s (1981) criterion, which compares the AVE of each construct with its squared inter-construct correlations. As shown in Table 2, all AVE values exceeded the corresponding squared correlations except for CP and IB, indicating adequate discriminant validity among three of the four constructs:

- PP: AVE = 0.71 (>0.56, 0.66, 0.64);
- CP: AVE = 0.75 (>0.66, 0.44, 0.76);
- SE: AVE = 0.85 (>0.66, 0.44, 0.50); and
- IB: AVE = 0.81 (>0.64, 0.76, 0.50).

**Table 2:** Results of discriminant validity testing.

	PP	CP	SE	IB
PP	0.71			
CP	0.56	0.75		
SE	0.66	0.44	0.85	
IB	0.64	0.76	0.50	0.81

PP, proactive personality; CP, creative personality; SE, student engagement; IB, innovative behavior. The gray shaded diagonal values indicate the average variance extracted (AVE) for each instrument.

However, because the CP and IB scales exhibited limited discriminant distinctiveness, discriminant validity was re-examined using the heterotrait–monotrait (HTMT) ratio calculated from the correlation coefficients reported in Table 3 (Henseler et al., 2015). The resulting HTMT value (*a*/√*b* × *c* = 0.682/√0.761 × 0.807 = 0.682/0.784 = 0.87) did not exceed the recommended cutoff of 0.90, indicating adequate discriminant validity between the two scales. In the preceding HTMT formula, *a* represents the heterotrait value, defined as the average of the correlations between measures of different latent constructs, whereas *b* and *c* represent the monotrait values, defined as the average of the correlations between measures within the same latent construct.

**Table 3:** Correlation coefficients, means, skewness, and kurtosis of measurement variables.

	(1)	(2)	(3)	(4)	(5)	(6)	(7)	(8)	(9)	(10)	(11)	(12)	(13)	(14)	(15)	(16)
PP1 (1)																
PP2 (2)	0.72**															
PP3 (3)	0.69**	0.70**														
Total (4)	0.88**	0.89**	0.91**													
SE1 (5)	0.60**	0.60**	0.62**	0.68**												
SE2 (6)	0.66**	0.66**	0.64**	0.73**	0.87**											
SE3 (7)	0.62**	0.67**	0.65**	0.72**	0.86**	0.92**										
Total (8)	0.66**	0.67**	0.66**	0.74**	0.94**	0.97**	0.96**									
CP1 (9)	0.59**	0.58**	0.55**	0.62**	0.54**	0.61**	0.57**	0.59**								
CP2 (10)	0.49**	0.54**	0.53**	0.58**	0.47**	0.52**	0.53**	0.52**	0.76**							
CP3 (11)	0.54**	0.57**	0.56**	0.62**	0.51**	0.56**	0.56**	0.57**	0.75**	0.77**						
Total (12)	0.58**	0.62**	0.59**	0.67**	0.55**	0.61**	0.60**	0.61**	0.92**	0.92**	0.92**					
IB1 (13)	0.62**	0.62**	0.60**	0.68**	0.58**	0.63**	0.62**	0.64**	0.66**	0.67**	0.68**	0.73**				
IB2 (14)	0.62**	0.59**	0.58**	0.66**	0.57**	0.62**	0.60**	0.61**	0.68**	0.67**	0.71**	0.75**	0.81**			
IB3 (15)	0.68**	0.60**	0.58**	0.68**	0.60**	0.66**	0.61**	0.65**	0.71**	0.66**	0.69**	0.75**	0.82**	0.80**		
Total (16)	0.68**	0.64**	0.63**	0.72**	0.63**	0.68**	0.65**	0.68**	0.74**	0.71**	0.79**	0.80**	0.92**	0.92**	0.95**	
Mean	3.48	3.79	3.74	3.68	3.51	3.55	3.71	3.59	3.67	3.91	3.78	3.79	3.69	3.69	3.60	3.65
SD	0.74	0.73	0.73	0.66	0.86	0.82	0.76	0.78	0.79	0.72	0.75	0.69	0.77	0.81	0.82	0.75
Kurtosis	0.47	1.37	1.22	1.66	0.53	0.29	1.25	0.72	0.48	2.55	1.54	2.06	1.18	0.48	0.31	0.92
Skewness	−0.09	−0.70	−0.69	−0.44	−0.47	−0.40	−0.67	−0.45	−0.46	−1.04	−0.77	−0.78	−0.70	−0.56	−0.43	−0.51

**p < 0.01, PP, proactive personality; CP, creative personality; SE, student engagement; IB, innovative behavior.

### 2.6 Examination of linearity and normality of indicators

To verify the linear relationships among the indicators of each latent variable, Pearson’s product–moment correlation coefficients were calculated. As shown in Table 3, correlations ranged from *r* = 0.51 to *r* = 0.79, all statistically significant at *p* < 0.01, confirming linear associations among the indicators.

To assess normality, skewness and kurtosis values were examined. As exhibited in Table 3, the absolute values of skewness ranged from 0.09 to 0.78 (below |2|), and kurtosis values ranged from 0.29 to 2.55 (below |4|). Therefore, all measurement indicators satisfied the assumptions of univariate normality.

## 3 Results

### 3.1 Descriptive statistics

On a 5-point scale, the mean scores for the four latent variables were as follows: PP = 3.68 (±0.66), CP = 3.79 (±0.69), SE = 3.59 (±0.78), and IB = 3.65 (±0.75). These results indicate that, on average, participants reported moderately high levels across all four constructs.

### 3.2 Relationships among variables (structural model validation)

Table 4 presents the product–moment correlation coefficients among the four latent variables, ranging from *r* = 0.66 to *r* = 0.87, all of which are statistically significant (*p* < 0.001).

**Table 4:** Correlation coefficient among latent variables (*n* = 618).

	PP	CP	SE
CP	0.75^***^		
SE	0.81^***^	0.66^***^	
IB	0.80^***^	0.87^***^	0.71^***^

***p < 0.001, PP, proactive personality; CP, creative personality; SE, student engagement; IB, innovative behavior.

To examine the hypothesized relationships among the latent variables, structural equation modeling (SEM) was conducted. This study adopted covariance-based structural equation modeling (CB-SEM), which is well suited for theory-driven research and the assessment of model fit. The application of CB-SEM presupposes a valid measurement model, sufficient sample size, and multivariate normality of the observed variables (Hair et al., 2017). All these assumptions were met in this study.

**Figure 1:**
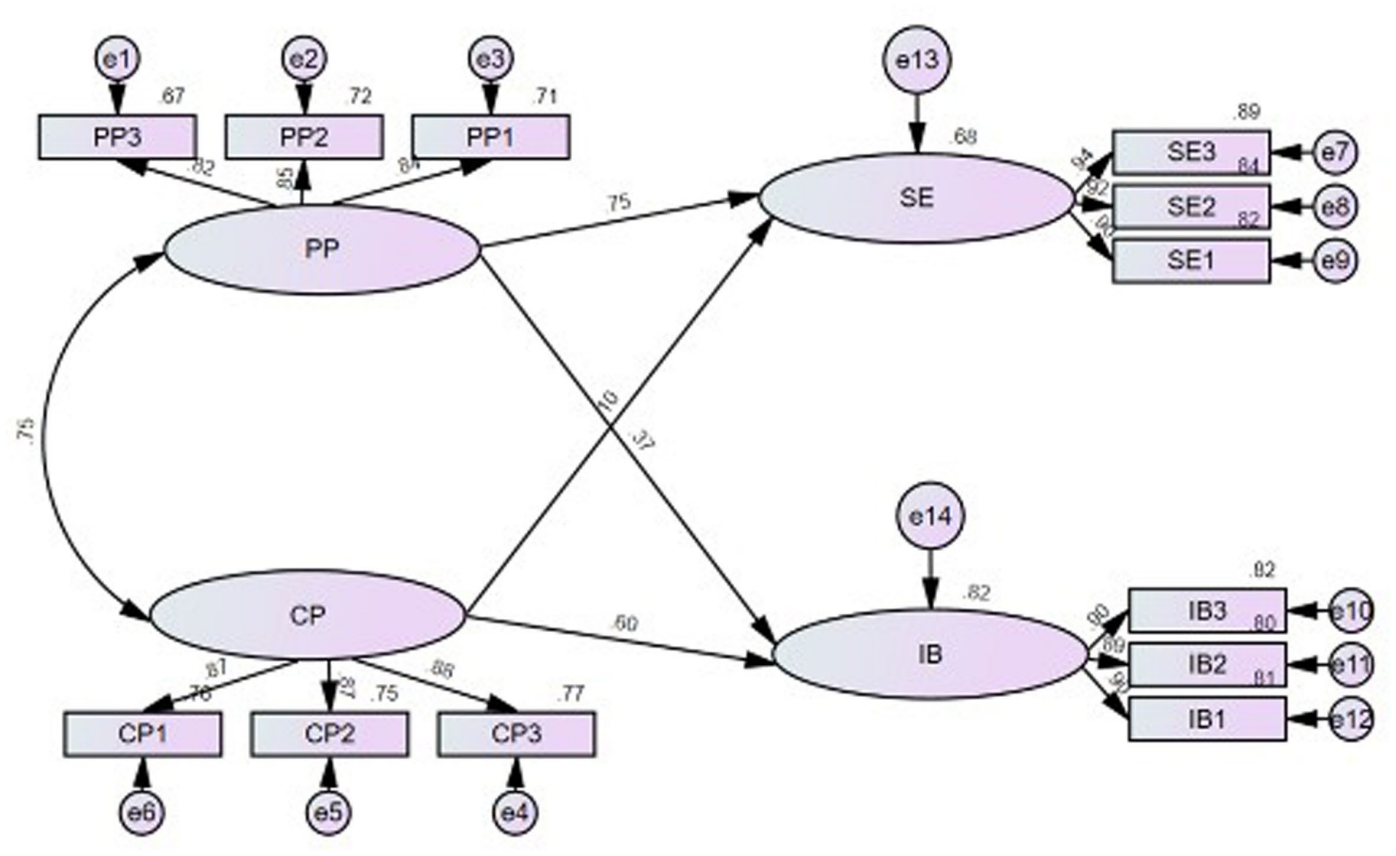
Structural model illustrating the relationships among proactive personality (PP), creative personality (CP), student engagement (SE), and innovative behavior (IB).

The overall model demonstrated a satisfactory level of fit, with the following indices: Tucker–Lewis Index (TLI) = 0.983, Standardized Root Mean Square Residual (SRMR) = 0.0179, and Root Mean Square Error of Approximation (RMSEA) = 0.054. These values meet the conventional criteria for good model fit, indicating that the structural model adequately represents the observed data (see Figure 1).

Table 5 summarizes the standardized path coefficients for the hypothesized relationships. The findings indicated that PP had a significant positive effect on both SE (*β* = 0.74, *p* < 0.001) and IB (*β* = 0.37, *p* < 0.001). CP also exerted direct effects on SE (*β* = 0.10, *p* > 0.05) and IB (*β* = 0.60, *p* < 0.001). The effect of CP on SE was not statistically significant, whereas its effect on IB was significant and positive. Comparative analysis of the standardized coefficients revealed that PP had a stronger influence on SE (*β* = 0.75) than on IB (*β* = 0.35) (*p* < 0.001). Conversely, CP had a significantly greater impact on IB (*β* = 0.60) than PP did (*β* = 0.35) (*p* < 0.01). PP was found to be a strong predictor of SE. This result appears theoretically sound because PP is conceptually defined as a behavioral tendency to proactively and actively change one’s environment and achieve goals (Bateman and Crant, 1993), whereas SE refers to the amount of effort invested in learning (Astin, 1984), the quality and intensity of initiative and enactment in learning activities (Fredricks et al., 2004), and the activeness and persistence of academic engagement (Schaufeli et al., 2002).

**Table 5:** Significance of structural path coefficients.

Path	*β*	*В*	95%CI	Cumulative SMC
PP (1) → SE	0.75^***^	0.90	0.63–0.87	
PP (2) → IB	0.37^***^	0.46	0.25–0.48	
CP (3) → SE	0.10	0.11	−0.05 to 0.24	68% (1 + 3)
CP (4) → IB	0.60^***^	0.67	0.48–0.72	82% (2 + 4)

SMC, squared multiple correlation *** *p* < .001.

By contrast, CP did not predict SE in the structural model. This is because PP and CP, which are highly correlated, were specified as covariates, and the shared variance attributable to the covariate was partialled out from the relationship between CP and SE. As shown in Table 4, both CP and SE (*r* = 0.66) and PP and SE (*r* = 0.81) show substantial correlations; however, the correlation between the covariates PP and CP is relatively large (*r* = 0.75). After controlling for this shared variance, the association between CP and SE was substantially reduced (*β* = 0.10). Because relationships between variables in a structural model are formed under the influence of other variables, the magnitude of simple correlations cannot be interpreted in an absolute manner. Accordingly, this result is best understood as indicating that although CP does predict SE, the strong correlations between PP and SE and between PP and CP imply that SE can be almost entirely predicted by PP alone.

The same logic applies to the relationships between CP and IB and between PP and IB. As shown in Table 4, the simple correlation between PP and IB is *r* = 0.80, and that between CP and IB is *r* = 0.87. After controlling for the effect of the covariate (PP–CP correlation, *r* = 0.75), the association between CP and IB became relatively stronger. This finding is contextually natural given that IB in higher education refers to intentions and behaviors related to applying new ideas, knowledge, and perspectives to real-life or academic contexts (Mei et al., 2015), whereas CP represents personality traits associated with creativity (Guilford, 1950; Lin, 2014; Wei, 2002; Jia et al., 2016) and tendencies toward creative behavior (Chen and Xiao, 2011; Wang and Wang, 2014).

Collectively, the results indicate that PP exerts positive effects on both SE and IB, with a stronger likelihood of influencing SE. By contrast, the independent effect of CP on SE is minimal, suggesting that the influence of CP on SE can be largely substituted by PP. Although PP and IB are correlated, this association is weaker than that between CP and IB, indicating that CP has a relatively stronger effect on IB than PP does. In this sense, PP has a stronger influence on SE than CP, whereas CP has a stronger influence on IB. However, because PP affects both SE and IB whereas CP does not affect SE, PP can substitute for CP in enhancing SE. Therefore, from a cost-effectiveness perspective, educational interventions aimed at enhancing PP may be more efficient than those focusing on CP. Considering these findings, Chinese universities should prioritize efforts to foster PP among college students.

## 4 Discussion and conclusion

This study investigated the interrelationships between PP and CP and examined their respective effects on SE and IB. Specifically, it analyzed the effects of PP on SE and IB while controlling for the mutual influence between PP and CP.

Individuals with high levels of PP tend to actively shape their environments rather than adapt passively, seize opportunities, and engage in goal-directed behavior. Consequently, they are more inclined to take risks, embrace challenges, exhibit curiosity, and seek novelty—traits conceptually aligned with CP. Prior research has shown that both PP and CP are positively associated with the Big Five traits of openness and extraversion (Fuller and Marler, 2009). Moreover, individuals with strong PP demonstrate creative leadership (Pan et al., 2018) and approach their tasks innovatively (Li et al., 2020), suggesting a positive association between PP and CP.

After controlling for their mutual influence, PP exerted a significant positive effect on SE (*β* = 0.75). This finding implies that individuals high in PP, who strive to overcome obstacles and achieve goals, are likely to invest considerable psychological and physical energy into their learning activities. Because SE was assessed through the dimensions of vigor, absorption, and dedication, it is reasonable to infer that PP exerts a strong influence on SE.

In contrast, CP showed a weak and statistically nonsignificant effect on SE (*β* = 0.10), suggesting that SE is primarily determined by PP. This interpretation is supported by the decline in the correlation between CP and SE—from *r* = 0.66 in the correlation matrix to *β* = 0.10 in the structural model—after controlling for shared variance between PP and CP. These findings align with prior research indicating that intelligence is correlated with learning outcomes, whereas creativity demonstrates weaker or no correlation (Getzels and Jackson, 1962; Benedek et al., 2014). Similarly, the threshold hypothesis suggests that the correlation between intelligence and creativity diminishes beyond a certain intelligence level (e.g., IQ 120; Jauk et al., 2013) and that IB is only weakly related to academic achievement (Benedek et al., 2014).

Even after controlling for CP, PP exerted a significant positive effect on IB (*β* = 0.35), suggesting that innovation requires not only creativity but also a proactive drive to implement and realize creative ideas. This finding is consistent with prior studies emphasizing the importance of proactive behavior in innovation (Seibert et al., 1999; Crant, 2000; Seibert et al., 2001; Parker et al., 2006; Liu, 2013; Cao and Xiang, 2014; Zhang et al., 2016; Pang and Wen, 2017; Zhou et al., 2020).

Conversely, when the influence of PP on CP was controlled, CP had a strong and significant positive effect on IB (*β* = 0.60), indicating that creativity is a critical determinant of innovation. The effect of CP on IB was significantly stronger than that of PP (*β* = 0.37), underscoring the central role of creativity in fostering innovation. These findings are consistent with prior studies that have identified a positive association between CP and IB (Zhou and George, 2001; Wang F., 2013; Wang X. D., 2013; Li et al., 2014; Song and Gu, 2015; Jia et al., 2016; Yu and Xiang, 2019).

Collectively, these findings suggest that PP is a strong antecedent of SE, while both PP and CP serve as important antecedents of IB. Enhancing PP may therefore promote greater academic engagement, whereas fostering both PP and CP may yield stronger creative performance and innovation.

Overall, PP contributes more substantially to SE than to IB, whereas CP contributes more strongly to IB than to SE. To prepare Chinese university students for success in the creativity- and innovation-driven Fourth Industrial Revolution, it is essential to cultivate both proactive and creative traits. This is particularly relevant given the collectivist and socialist cultural context in which Chinese students develop. Because PP and CP are not innate traits but can be strengthened through personal experience, deliberate effort, and improved educational practices, enhancing these characteristics represents a meaningful pathway to academic and creative development.

Fostering PP and CP among Chinese university students cannot be achieved solely through isolated or short-term programs. Rather, it requires fundamental changes in the awareness of educational policymakers, faculty members, and students themselves, as well as a departure from long-standing collective educational practices that have been repeatedly institutionalized in Chinese higher education.

Based on the present findings, several policy-level directions can be proposed as immediate priorities. First, higher education policies should encourage a systematic shift from traditional lecture-based and teacher-centered instructional methods toward discussion-oriented and problem-solving–based pedagogical approaches. Such instructional reforms can provide students with greater opportunities for autonomous thinking and active engagement, which are essential for the development of PP and CP. Second, curriculum policies should gradually move away from rigid compulsory course structures toward more flexible, elective-based systems, allowing students to exercise greater choice and self-direction in their academic pathways. Third, residential policies should be revised so that on-campus dormitory living is no longer mandatory for all students; instead, students who wish to do so should be permitted to reside off campus, thereby promoting autonomy and personal responsibility in daily life.

Together, these policy directions illustrate how the results of the present study can be translated into concrete educational policies aimed at enhancing students’ psychological development and individual agency within the context of Chinese higher education.

## 5 Limitations and contributions

This study has several limitations that warrant consideration. First, data were collected from multiple universities, resulting in a multilevel dataset. Ideally, such data should be analyzed using multilevel SEM or other statistical procedures that account for hierarchical structures. However, these methods were not applied in this analysis because respondents’ university affiliations were intentionally not collected at the survey design stage. This decision was made to encourage honest responses from students and to avoid potential ethical concerns associated with identifying institutional affiliations. Consequently, if the relationships among the latent variables differed systematically across universities, these variations were not captured.

Second, the discriminant validity between the measurement instruments for CP and IB warrants caution. Although conceptually distinct, the relatively high correlation between these constructs suggests limited statistical differentiation. This overlap likely reflects the conceptual proximity of creativity and innovation, which, while distinct psychological phenomena, are inherently related.

Despite these limitations, this study makes several theoretical and practical contributions by extending and integrating prior research. Previous studies have consistently demonstrated that PP is a significant predictor of CP and also serves as an important predictor of SE (Schaufeli et al., 2002; Zhang et al., 2008). However, the present study demonstrates that when PP and CP are simultaneously considered, PP is sufficiently robust to substitute for CP in predicting SE. This finding highlights the dominant role of PP in explaining academically oriented psychological outcomes, thereby refining our understanding of the relative predictive power of these two personality constructs.

In addition, this study confirmed that PP significantly predicts IB, providing further empirical support for prior findings that link PP to behaviors involving the generation of new ideas (Gu and Peng, 2010). Concurrently, the strong predictive effect of CP on IB corroborates previous research demonstrating the close association between CP and innovative outcomes (Wang F., 2013; Wang X. D., 2013; Li et al., 2014; Song and Gu, 2015; Gao et al., 2016; Jia et al., 2016; Yu and Xiang, 2019).

Moreover, the finding that CP more strongly predicts IB—an outcome closely related to creativity—than SE, which is more directly associated with academic achievement, indirectly supports the intelligence threshold theory. This theory posits that although intelligence and creativity are related, intelligence beyond a certain level (e.g., IQ 120) is no longer strongly associated with creativity (Benedek et al., 2014; Jauk et al., 2013). By empirically distinguishing the differential roles of PP and CP across SE, IB, and academic achievement, this study contributes to the theoretical refinement of personality–outcome relationships in higher education contexts.

Beyond its theoretical implications, this study offers important practical contributions. The findings suggest that for China to achieve further societal and economic development, higher education should foster university students who move beyond collectivist and conformist modes of thinking toward greater creativity and innovation. To this end, it is necessary to comprehensively reform educational systems, policies, and curricular practices that may inhibit the development of students’ PP and CP. In this regard, the present study provides meaningful implications for educational practice and policy aimed at cultivating innovative human capital.

## Data Availability

Publicly available datasets were analyzed in this study. This data can be found here: https://osf.io/78mrp/overview.

## Footnotes

1 www.gov.cn, November 14, 2022.

2
www.gov.cn
