# Supplementary material for: Proactive and creative personality as predictors of student engagement and innovative behavior among Chinese university students
Source: Front Psychol. 2026 Mar 2;17:1752174. doi: 10.3389/fpsyg.2026.1752174 (PMC12990132; doi:10.3389/fpsyg.2026.1752174)
Supplement: Supplementary file 1 [file Supplementary_file_1.docx]

Supplementary Material – Appendix A: Questionnaire

Dear Participants,
This survey aims to examine variables associated with students’ academic engagement and innovative behavior. Your sincere and thoughtful responses are essential to the validity and reliability of this research. The estimated time required to complete the questionnaire is approximately five minutes. Participation is voluntary, and you may withdraw at any time without any negative consequences. All responses will remain strictly anonymous, and the collected data will be used exclusively for academic research purposes. Thank you for your valuable time and cooperation.

1. Consent to Participate
Please indicate your willingness to participate in this survey:
a. Yes, I agree to participate.
b. No, I do not agree to participate.

2. Gender
a. Male
b. Female

3. Year of Study
a. Freshman
b. Sophomore
c. Junior
d. Senior

Instructions:
Please read each statement carefully and select the number that best reflects your level of agreement.
a. Strongly disagree (1)
b. Disagree (2)
c. Neutral (3)
d. Agree (4)
e. Strongly agree (5)

Proactive Personality
1. I am constantly on the lookout for new ways to improve my life.
2. Wherever I have been, I have been a powerful force for constructive change.
3. Nothing is more exciting than seeing my ideas turn into reality.
4. If I see something I don’t like, I fix it.
5. No matter what the odds, if I believe in something, I will make it happen.
6. I love being a champion for my ideas, even against others’ opposition.
7. I excel at identifying opportunities.
8. I am always looking for better ways to do things.
9. If I believe in an idea, no obstacle will prevent me from making it happen.
10. I can spot a good opportunity long before others can.

Student Engagement
1. When I’m studying, I feel mentally strong.
2. I can continue for a very long time when I am studying.
3. When I study, I feel like I am bursting with energy.
4. When studying, I feel strong and vigorous.
5. When I get up in the morning, I feel like going to class.
6. I find my studies to be full of meaning and purpose.
7. My studies inspire me.
8. I am enthusiastic about my studies.
9. I am proud of my studies.
10. I find my studies challenging.
11. Time flies when I’m studying.
12. When I am studying, I forget everything else around me.
13. I feel happy when I am studying intensively.
14. I can get carried away by my studies.

Innovative Behavior
1. When I encounter difficulties, I can always think of alternative ways to solve them.
2. I have many new ideas.
3. I tend to create new methods rather than merely improve existing ones.
4. I can handle several new ideas and problems at the same time.
5. When facing old problems, I often have new perspectives.
6. I often help others generate new ideas.
7. I need frequent changes and stimulation to come up with new ideas.
